# Improved characterization of infarct heterogeneity from high resolution T_1_^*^ maps using compressed sensing and temporal PCA with weighted total variation

**DOI:** 10.1186/1532-429X-17-S1-W33

**Published:** 2015-02-03

**Authors:** Li Zhang, Prashant Athavale, Venkat Ramanan, Jennifer Barry, Garry Liu, Nilesh R Ghugre, Mihaela Pop, Graham A Wright

**Affiliations:** 1 Medical Biophysics, University of Toronto, Toronto, ON, Canada; 2 Schulich Heart Research Program and Physical Sciences Platform, Sunnybrook Research Institute, Toronto, ON, Canada; 3 Department of Mathematics, University of Toronto, Toronto, ON, Canada

## Background

Characterization of infarct heterogeneity can inform therapeutic strategies for arrhythmia management of patients with prior myocardial infarction (MI). Multi-contrast late-enhancement (MCLE) [1] images along the signal relaxation curve, acquired in a breath-hold ECG-gated scan, offer better visualization of MI than IR-GRE. A T_1_^*^ map and steady state image M_ss_ are then used to quantitatively characterize infarct heterogeneity. However, motion and constrained imaging durations typically yield low spatial resolution images and blurry anatomical borders in infarcted regions on a T_1_^*^ map. This work explores the feasibility of accelerating the MCLE acquisition using Compressed Sensing and temporal Principal Component Analysis (CS-tPCA) to achieve higher spatial resolution images and T_1_^*^ maps, while preserving anatomical edges using weighted total variation (TV) in the reconstruction, to eventually improve infarct heterogeneity characterization.

## Methods

Reperfused MI was induced in 2 pigs by complete occlusion of the LAD artery for 90 min. At four weeks, the animals were imaged in vivo using MCLE after injecting 0.2 mmol/kg Gadolinium-DTPA. The fully sampled dataset of each slice was acquired, using a four-channel anterior cardiac coil array, over a 24-s breath-hold to achieve an in-plane spatial resolution of 1.25 mm (monitored HR = 92 beats/min), and was then retrospectively undersampled in the outer k-space region to yield a net acceleration factor of 2.67. With PCA performed on a low-rank Casorati matrix formed from the central region of k-t space, the principal components (PC) of the temporal signal evolution were extracted. The weighted TV regularization was applied in the CS framework to reconstruct the PC coefficient maps from undersampled datasets. The MCLE images were then obtained by a coefficient-weighted sum of PCs and used to obtain the T_1_^*^ and M_ss_ maps from a non-linear least squares parameter fitting, which were then used in a fuzzy c-means clustering algorithm for tissue classification. For comparison, an alternative reconstruction, REPCOM [2], was also implemented.

## Results

**Figure 1:**
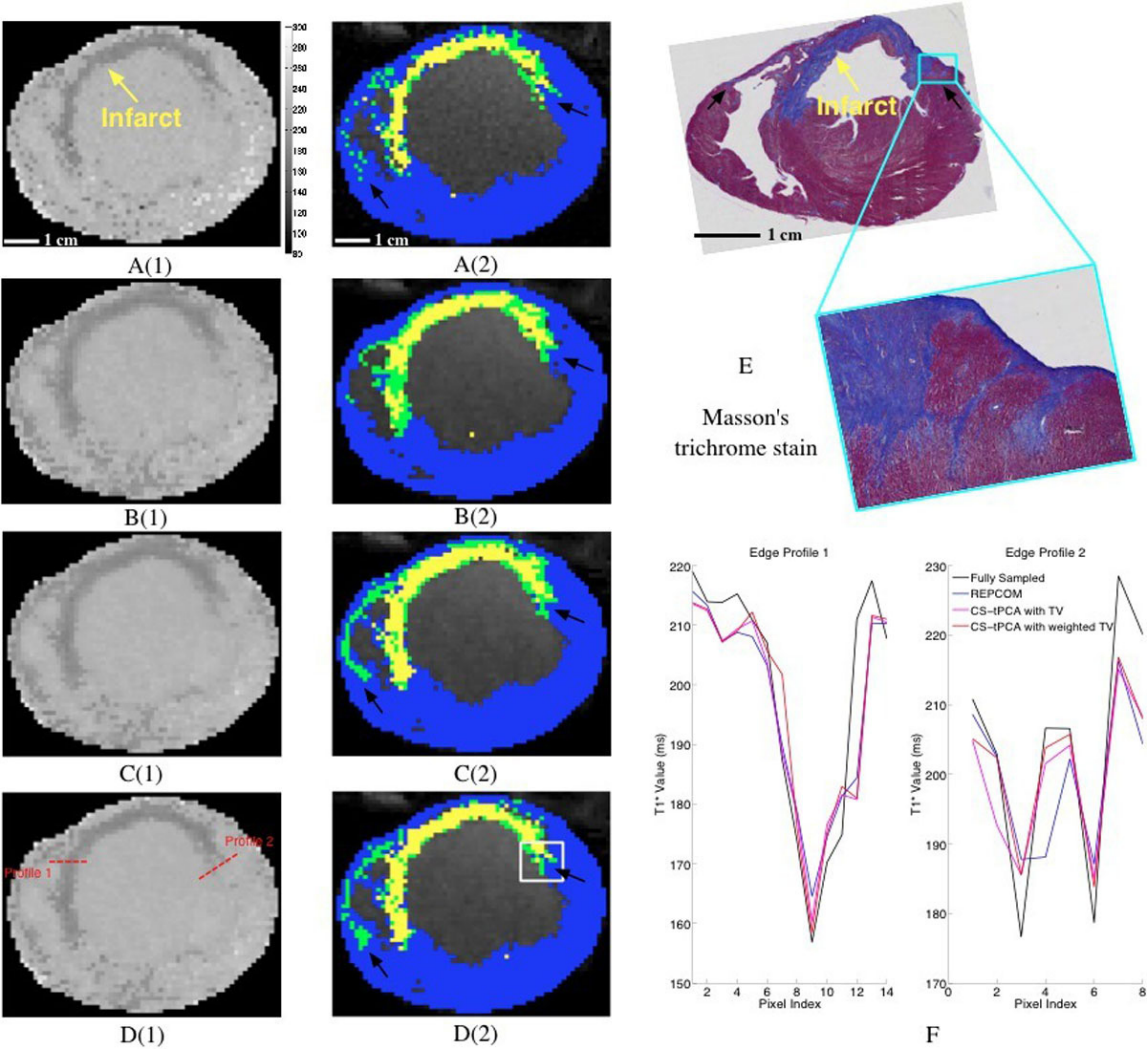
The T_1_^*^ maps and classification results of a representative slice in the short axis view. A(1), B(1), C(1), D(1) are T_1_^*^ maps reconstructed from fully sampled datasets, REPCOM, CS-tPCA with TV and CS-tPCA with weighted TV. The dark rim along the wall with smaller T1^*^ values indicates the infarct region. A(2), B(2), C(2), D(2) are classification results from fully sampled datasets, REPCOM, CS-tPCA with TV and CS-tPCA with weighted TV. The classification results are overlaid onto the corresponding anatomical images. Blue, green and yellow indicate healthy myocardium, heterogeneous infarct and scar. Pixels classified as blood are excluded. Histology image E includes a magnified area corresponding to the white box in D(2), and matches the MCLE slice by visual inspection on the anatomical geometry and critical landmarks such as trabeculations. F shows two profiles from the various methods, plotted across the red dashed lines indicated in D(1).

**Figure 2:**
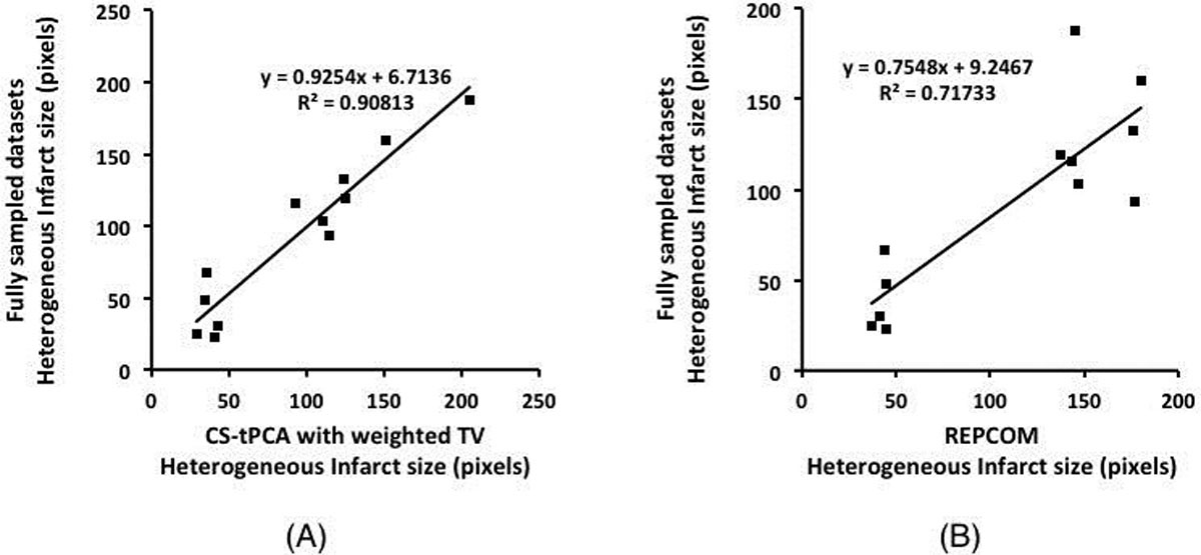
Correlations in heterogeneous infarct size between CS-tPCA with weighted TV and fully sampled datasets (A), and between REPCOM and fully sampled datasets (B) for all the slices with obvious infarcts for two pigs in the in-vivo study.

From Fig. 1, the T_1_* map from CS-tPCA with weighted TV presents the most sharply defined edges in heterogeneous infarct regions; the classification result from CS-tPCA with weighted TV is more consistent with that from the fully sampled dataset and the histology image, particularly in terms of the features indicated by the arrows. From Fig. 2, there is good agreement in the heterogeneous infarct size between the fully sampled dataset and CS-tPCA with weighted TV, while REPCOM yields weaker agreement with the fully sampled dataset.

## Conclusions

We successfully demonstrated that improving characterization of infarct heterogeneity is feasible in a high-spatial-resolution acquisition using compressed sensing and temporal PCA, with edge preservation in infarcted regions using weighted TV in the reconstruction.

## Funding

GE Healthcare and CIHR grant.
